# EasyDetectDisease: An Android App for Early Symptom Detection and Prevention of Childhood Infectious Diseases

**DOI:** 10.2196/12664

**Published:** 2019-05-14

**Authors:** Mahvish Ponum, Osman Hasan, Saadia Khan

**Affiliations:** 1 School of Electrical Engineering and Computer Science National University of Sciences and Technology Islamabad Pakistan; 2 Department of Pediatrics The Children’s Hospital & Institute of Child Health Multan Multan Pakistan

**Keywords:** infectious diseases, mHealth, causes of death

## Abstract

**Background:**

Infectious diseases often lead to death among children under 5 years in many underdeveloped and developing countries. One of the main reasons behind this is an unawareness of disease symptoms among mothers and child caregivers. To overcome this, we propose the EasyDetectDisease mobile health app to educate mothers about the early symptoms of pediatric diseases and to provide them with practical advice for preventing the spread of such diseases in children under 5 years. The EasyDetectDisease app includes detailed knowledge of infectious diseases, including the corresponding symptoms, causes, incubation period, preventive measures, nutritional guidelines such as breastfeeding, video tutorials of child patients, and video guidelines by pediatric health experts to promote child health. It also provides information on the diagnosis of the infectious diseases based on symptoms.

**Objective:**

The objective of this study was to evaluate the usability (eg, ease of use, easy detection of disease, functionality, and navigation of interfaces) of the EasyDetectDisease app among mothers of children under 5 years of age.

**Methods:**

Two health sessions, held in Pakistan, were used to evaluate the usability of EasyDetectDisease by 30 mothers of children under 5 years. The app was evaluated based on various quantitative and qualitative measures.

**Results:**

The participating mothers confirmed that they were able to diagnose diseases accurately and that after following the instructions provided, their children recovered rapidly without any nutritional deficiency. All participating mothers showed an interest in using the EasyDetectDisease app if made available by governmental public health agencies, and they suggested its inclusion in all mobile phones as a built-in health app in the future.

**Conclusions:**

EasyDetectDisease was modified into a user-friendly app based on feedback collected during the usability sessions. All participants found it acceptable and easy to use, especially illiterate mothers. The EasyDetectDisease app proved to be a useful tool for child health care at home and for the treatment of infectious diseases and is expected to reduce the mortality rate of children under 5 years of age.

## Introduction

### Overview

Child health care is one of the foremost priorities in the world. However, it is an undeniable truth that millions of children’s lives are affected each year by disease. Infectious diseases have been regarded as a major cause of morbidity and mortality in children under 5 years of age, according to a recent report by the World Health Organization (WHO) [1], 83% of the global deaths of children under 5 years are caused by infectious diseases and malnutrition. According to another recent WHO report [2], around 5.6 million (60%) children died before reaching their fifth birthday in 2016 [2], out of which 3.6 million deaths happened in 10 South Asian and African countries. Out of these 3.6 million children, almost half of the deaths occurred in 5 countries (ie, Nigeria, the Democratic Republic of the Congo, India, Ethiopia, and Pakistan). Pakistan is one of the developing countries that is facing the challenge of a high child mortality rate because of infectious diseases. The mortality rate was 78.8 in 2016 [3], with a 40% ratio of infectious diseases in Pakistan [4].

A mother is considered to be a sick child's first health care provider at home. Various studies have been conducted in Pakistan to show a strong correlation between maternal education and lower child mortality [5]. These studies examined health-seeking behaviors, specifically the education level of the mother and its effects on child mortality [5]. It was identified that the behavior of mothers who had a better understanding of childhood diseases and were accustomed to using modern health care services was quite distinct from those who had no knowledge of childhood diseases. Therefore, maternal education can be regarded as an important contributor to child health development and can be used to overcome the abovementioned issues.

In Pakistan, however, to the best of our knowledge, very little effort is being made to educate mothers about pediatric infectious and noninfectious diseases. It has been found that a lack of child health awareness, inadequate maternal education, improper access to medical facilities, and a lack of exposure to smart technology and mobile health (mHealth) tools are some of the key causes behind child mortality in Pakistan [6-8]. In this paper, we focused on maternal education, child care awareness, and provision of mHealth tools as potential solutions for reducing the mortality rate of children under 5 years. In particular, we present the mHealth app EasyDetectDisease to educate mothers about childhood infectious diseases. The EasyDetectDisease app provides information on the diagnosis of infectious diseases based on symptoms, the prevention of the spread of such diseases, nutritional recommendations for children under 5 years, as well as sick patients’ tutorials and guidelines formulated by pediatricians.

### Global Death Estimates for Children Under 5 Years by Cause and Sex (2000-2015)

This subsection provides the estimated statistics of global deaths by cause and sex of children under 5 years while focusing on the statistics for Pakistan. Updated data of children under 5 years has been retrieved from the WHO and United Nations International Children’s Emergency Fund (UNICEF) websites; specific information has been extracted and presented in tables.

According to the WHO’s Global Health Observatory, globally, 15,000 children died every day in 2016 (5.6 million per year) [9] and 16,000 died per day in 2015 (5.9 million per year) [10]. Globally, under 5 years deaths declined to 5.6 million in 2016 from 12.7 million in 1990 [11]. Table 1 shows the figures for deaths by cause and sex of children under 5 years since 2000 (see Multimedia Appendix 1 for complete data).

**Figure 1:**
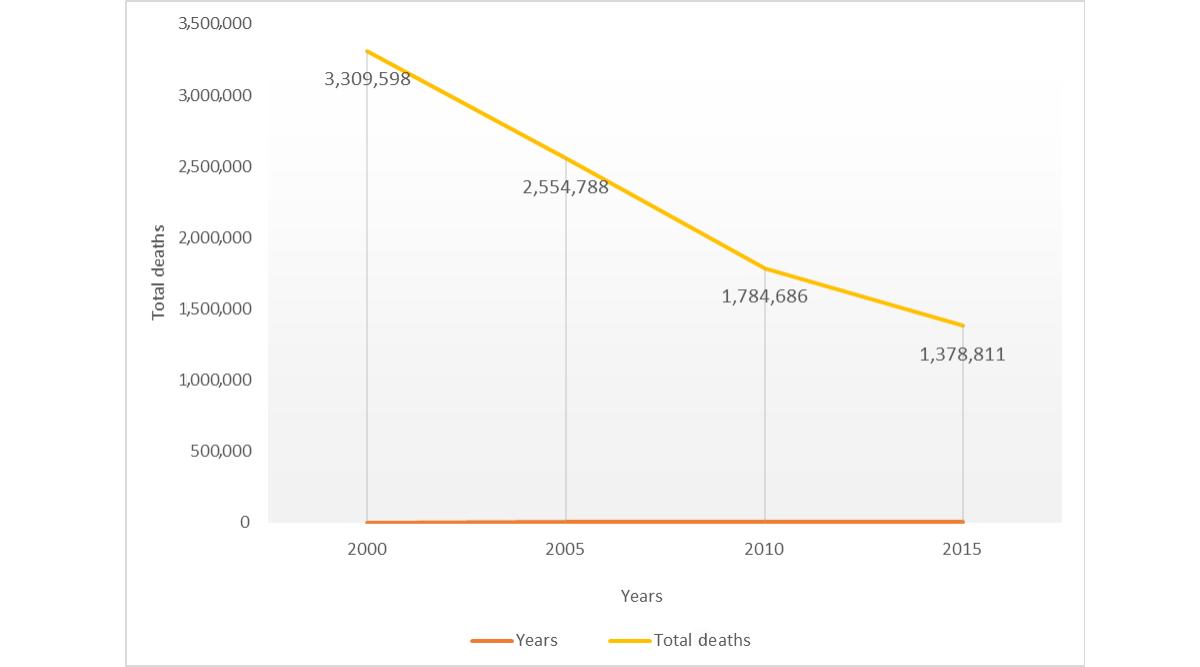
Decline in deaths due to infectious and parasitic diseases among children under 5 years of age.

It can be observed from Table 1 that the ratio of deaths due to infectious diseases is higher than other kinds of diseases. The total number of deaths caused by infectious and parasitic diseases declined from 3,309,598 in 2000 to 1,378,811 in 2015 [12]. Figure 1, based on the data from Table 1, shows the decline in deaths caused by infectious and parasitic diseases.

Globally, most countries achieved remarkable progress in the reduction of deaths among children under 5 years during the period of 2000 to 2015. However, it is still expected to reach 5 million deaths by 2025 [13], according to the WHO, and 97% of them would be in the developing regions of the world, including Pakistan, and due to infectious and parasitic diseases such as pneumonia, diarrhea, and inappropriate nutritional conditions [11]. If the current trends continue and appropriate measures are not taken, it is expected to reach a total of 69 million global deaths, that is, 4.6 million deaths per year, by 2030 [3]. Moreover, only 5 countries would be responsible for more than the half of these deaths, that is, India for 17% of deaths, Nigeria for 15% of deaths, Pakistan for 8% of deaths, the Democratic Republic of the Congo for 7% deaths, and Angola for 5% of deaths [3].

**Table 1:** Deaths by cause and sex of children under 5 years of age (2000-2015): global statistics.

Year			2000		2005		2010		2015	
			Male	Female	Male	Female	Male	Female	Male	Female
Population (thousands)			307,365	288,056	314,316	293,895	327,383	305,647	340,696	318,471
**All causes**			3,125,021	3,023,960	2,573,082	2,443,545	2,067,721	1,943,638	1,726,318	1,582,772
	**Communicable, maternal, perinatal, and nutritional conditions**		2,613,990	2,573,705	2,107,444	2,037,486	1,606,145	1,538,904	1,293,035	1,209,656
		Infectious and parasitic diseases	1,675,701	1,633,897	1,296,962	1,257,826	906,321	878,365	705,280	673,531
		Respiratory infections	703,758	729,592	598,657	595,634	495,105	486,014	389,846	370,367
		Maternal conditions	0	0	0	0	0	0	0	0
		Neonatal conditions	122,311	115,293	112,414	104,739	108,504	101,210	105,356	98,276
		Nutritional deficiencies	112,220	94,924	99,411	79,287	96,215	73,314	92,553	67,482
	**Noncommunicable diseases**		296,596	285,550	269,893	256,698	255,631	240,535	246,338	227,590
		Malignant neoplasm	20,978	16,204	19,968	15,572	20,604	15,684	21,155	16,042
		Other neoplasm	1557	1404	1410	1340	1749	1551	1779	1591
		Diabetes mellitus	1343	1399	1149	1184	1015	1041	874	885
		Endocrine, blood, and immune disorders	35,753	31,327	34,870	29,505	35,466	28,493	37,397	27,851
		Mental and substance use disorders	0	0	0	0	0	0	0	0
		Neurological conditions	11,423	8292	10,747	8086	10,085	7995	9545	7916
		Sense organ diseases	0	0	0	0	0	0	0	0
		Cardiovascular diseases	31,539	25,723	27,031	21,784	23,824	19,130	20,714	16,764
		Respiratory diseases	31,611	28,215	25,740	21,118	22,662	17,819	20,490	15,333
		Digestive diseases	18,479	20,786	16,015	19,123	13,979	17,066	12,166	15,005
		Genitourinary diseases	12,058	10,541	10,995	9226	10,364	8486	9823	7743
		Skin diseases	2123	2994	2084	3128	2119	3473	2307	3897
		Musculoskeletal diseases	0	0	0	0	0	0	0	0
		Congenital anomalies	119,252	130,087	109,316	117,983	103,514	111,222	99,896	105,862
		Oral conditions	0	0	0	0	0	0	0	0
		Sudden infant death syndrome	10,479	8579	10,569	8647	10,250	8576	10,192	8700
	**Injuries**		214,436	164,704	195,745	149,362	205,945	164,199	186,944	145,526
		Unintentional injuries	203,682	154,364	187,073	141,177	196,940	155,988	176,700	136,424
		Intentional injuries	10,754	10,340	8672	8185	9005	8212	10,244	9102

#### Death Estimates for Children Under 5 Years by Cause in Pakistan (2000-2015)

**Figure 2:**
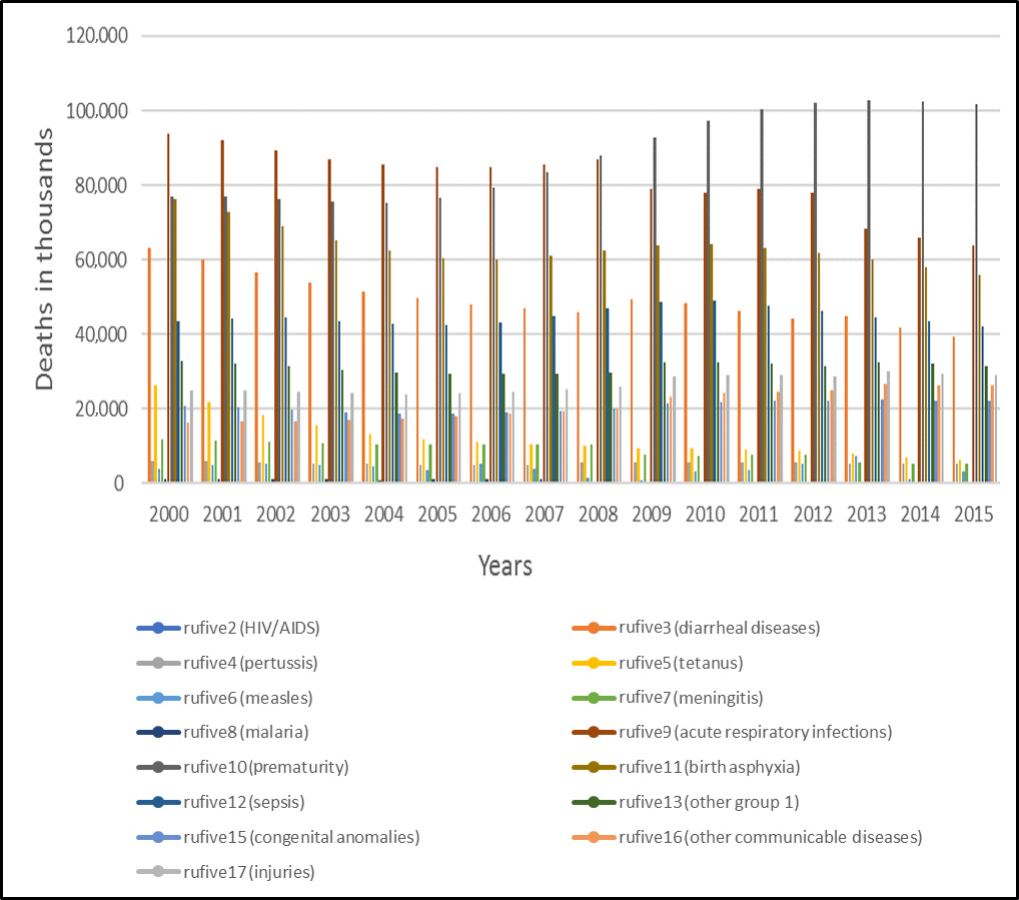
Estimates for causes of death among children under 5 years of age in Pakistan (2000-2015).

Pakistan is ranked sixth among 22 high disease–burden countries and is facing the challenge of high communicable diseases with a 40% ratio [4]. The leading causes of under 5 years deaths include premature birth, pneumonia, diarrhea, and malaria. The total number of deaths caused by various diseases is depicted in Figure 2 to assess the top 20 most dangerous infectious diseases [14].

**Figure 3:**
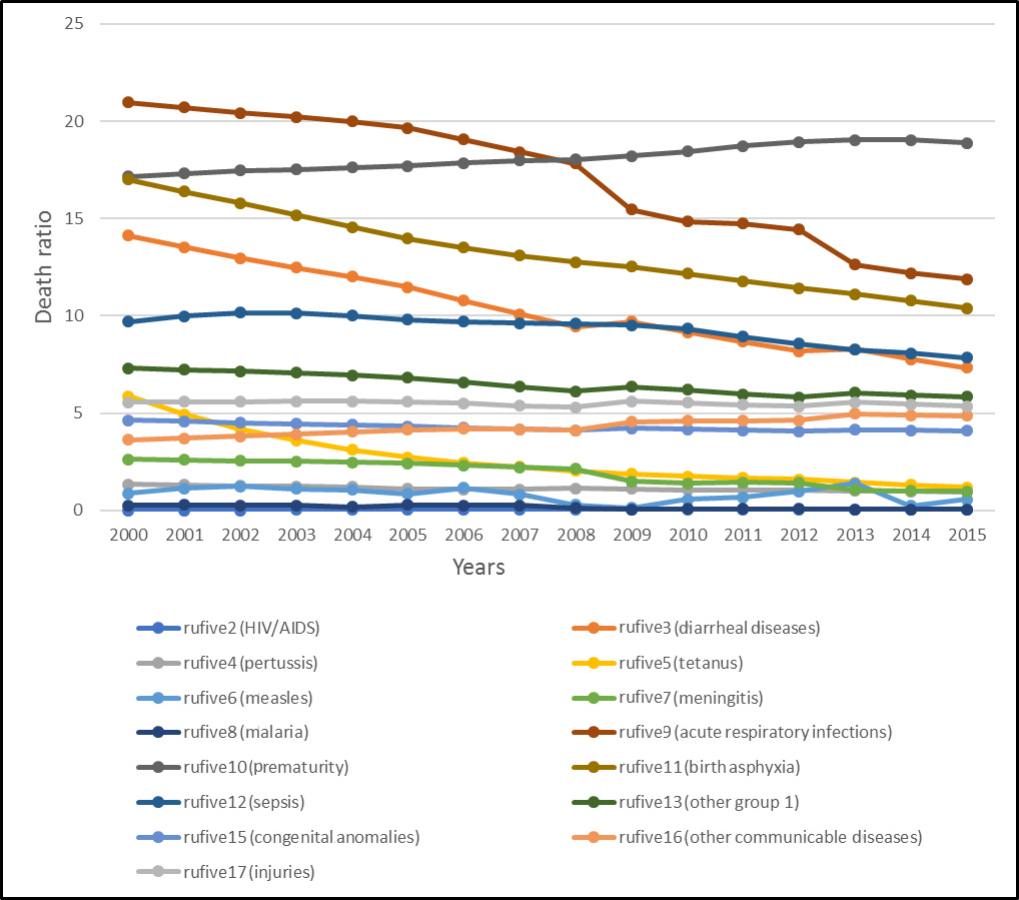
Death rate of children under 5 years of age due to various causes in Pakistan (2000-2015).

In Figure 2, we can see that the ratio of deaths due to acute respiratory infections (cause 9) was quite high in 2000. This decreased gradually until 2008. Prematurity (cause 10) increased rapidly after 2008, and even now, it is the most dominant cause of death. Birth asphyxia (cause 11) was high in 2002, that is, ranked third, but it was controlled by 2006. After 2006, birth asphyxia again prevailed and the number of deaths associated with it continued to increase until 2012. The number of deaths due to diarrheal diseases (cause 3) increased by the mid 2000s but was controlled in 2008, although a minor increase was seen in 2009 and 2010 and it persisted as a major cause of under 5 years child mortality in Pakistan. Sepsis (cause 12) was ranked as the fifth major cause of death by 2005, and then, the number of corresponding deaths increased higher than diarrheal diseases in 2015. Deaths due to cause 13 (other group 1) diseases, cause 17 (injuries), cause 16 (other communicable diseases), cause 15 (congenital anomalies), cause 5 (tetanus), cause 7 (meningitis), cause 4 (pertussis), cause 6 (measles), cause 8 (malaria), and cause 2 (HIV/AIDS) were prominent in Pakistan, as reported by the WHO. The death rate of children under 5 years (variable name “rufive”; the data for rufive was retrieved from the WHO website and is shown in Table 2 and the figures) is depicted in Figure 3.

**Table 2:** Deaths by cause and sex of children under 5 years of age (2000-2015): statistics for Pakistan.

Year			2000		2005		2010		2015	
			Male	Female	Male	Female	Male	Female	Male	Female
Population (thousands)			10,399	9703	10,441	9610	11,609	10,791	12,792	11,871
**All causes**			264.1	233.2	234.7	201	253.5	216.7	233.2	195.7
	**Communicable, maternal, perinatal, and nutritional conditions**		229.7	205.9	200.1	174.9	210.8	184.2	189.8	162.2
		Infectious and parasitic diseases	62.8	64.5	47.3	47.2	42.6	44.6	33.6	35.3
		Respiratory infections	43	50.9	40.5	44.3	37	41	31.1	32.8
		Maternal conditions	0	0	0	0	0	0	0	0
		Neonatal conditions	122.5	89.4	111.3	82.4	129.6	97.2	123.2	92.6
		Nutritional deficiencies	1.3	1.1	1.1	1	1.6	1.4	1.8	1.4
	**Noncommunicable diseases**		21.1	15.8	20.6	14.7	26.5	19.6	27.7	20.4
		Malignant neoplasm	0.9	0.5	1	0.6	1.3	0.8	1.6	1
		Other neoplasm	0.1	0.1	0.1	0.1	0.1	0.2	0.2	0.3
		Diabetes mellitus	0.1	0.1	0.1	0.1	0.1	0.2	0.1	0.2
		Endocrine, blood, and immune disorders	0.9	0.9	0.9	0.9	1.4	1.4	1.6	1.6
		Mental and substance use disorders	0	0	0	0	0	0	0	0
		Neurological conditions	0.5	0.4	0.6	0.5	0.8	0.8	0.9	1
		Sense organ diseases	0	0	0	0	0	0	0	0
		Cardiovascular diseases	0.9	0.2	1	0.2	1.3	0.3	1.4	0.3
		Respiratory diseases	4.7	2	5.2	2.1	6.4	2.7	6.6	2.8
		Digestive diseases	1.1	0.6	1	0.5	1.5	0.8	1.4	0.9
		Genitourinary diseases	0.7	0.3	0.8	0.3	1.1	0.5	1.2	0.5
		Skin diseases	0.1	0.4	0.1	0.5	0.2	0.8	0.2	1
		Musculoskeletal diseases	0	0	0	0	0	0	0	0
		Congenital anomalies	10.8	9.9	9.9	8.7	11.5	10.3	11.7	10.2
		Oral conditions	0	0	0	0	0	0	0	0
		Sudden infant death syndrome	0.4	0.4	0.6	0.5	0.7	0.7	0.9	0.8
	**Injuries**		13.4	11.5	13.3	10.8	16.2	12.9	15.7	13.2
		Unintentional injuries	12.8	11	12.9	10.4	15.3	12	14.7	12.1
		Intentional injuries	0.6	0.6	0.4	0.4	0.9	0.9	1	1

#### Pakistan’s Estimated Under 5 Years Deaths by Cause and Sex (2000-2015)

According to the WHO, deaths among children under 5 years of age in Pakistan because of contagious diseases are higher than those linked to noncontagious diseases [11]. In Pakistan, the number of deaths caused by infectious diseases was 352,000 in 2015 [10]. Table 2 shows the figures for deaths of Pakistani children under 5 years by causes and sex (see Multimedia Appendix 1 for complete data).

**Figure 4:**
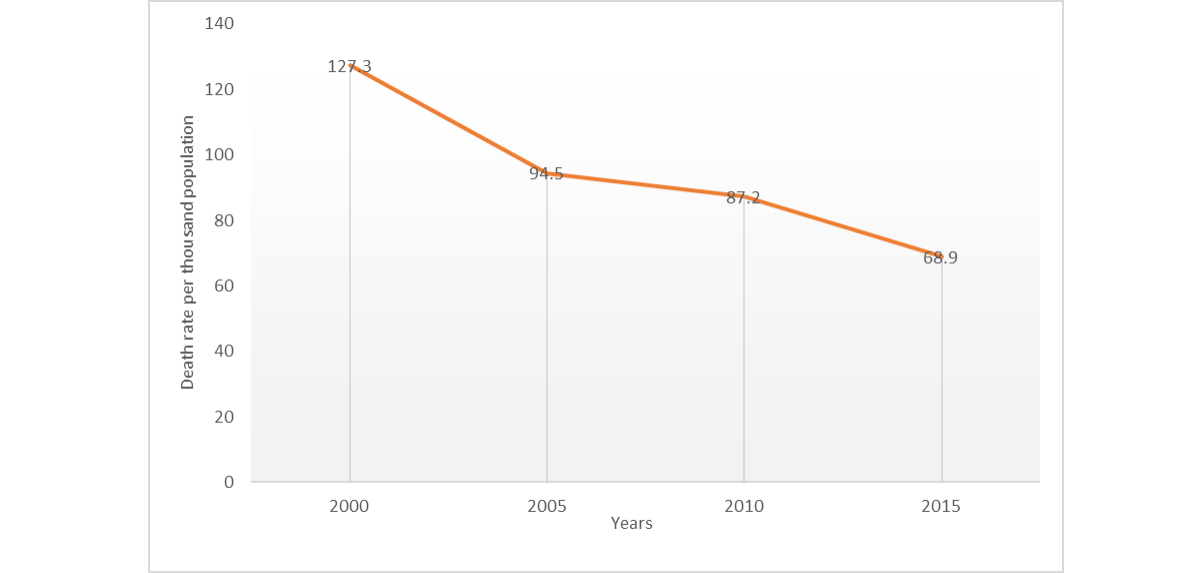
Decline in child death rate caused by infectious and parasitic diseases in Pakistan.

We can see from Table 2 that the infectious disease death ratio is higher than other categories of diseases. The total death rate due to infectious and parasitic diseases declined from 127.3 per 1000 children in 2000 to 68.9 per 1000 children in 2015 [12]. Figure 4, based on data from Table 2, shows a decline in the death rate of children in Pakistan due to infectious and parasitic diseases.

Pakistan has achieved remarkable progress in the reduction of deaths among children under 5 years during the period of 2000 to 2015, as shown in Figure 4. Despite achieving progression in the fourth goal (*Reduce Child Mortality*) of Millennium Development Goals, 4.6 million child deaths per year is projected for the period of 2016 to 2030, and Pakistan is expected to be responsible for 8% of these deaths [3]. These numbers are quite alarming and require preventive measures to be taken.

### Related Works and Motivation

mHealth is an area of electronic health that deals with health aids via mobile phones [15-16]. The most common practice of mHealth is the use of smartphones to educate users about health care services [17]. To the best of our knowledge, no research focusing on minimizing the mortality of children under 5 years using mHealth-based maternal education in Pakistan has been conducted before.

It has been observed that most Pakistani mothers are not aware of diseases, their prevention, and child care at home [18]. Mothers in Pakistan mostly use homemade remedies instead of providing proper medical care to their children. Such actions often lead to disease severity or even death of the child in the worst-case scenarios. To promote child health, the government of Pakistan periodically initiated various health care programs. In 2010, working with the Ministry of Health, nongovernmental organizations (NGOs) and UNICEF launched Maternal, Neonatal and Child Health [19] and the national Expanded Program on Immunization programs to overcome the problems of diseases, to monitor the health of mothers and children, and to promote child development [20]. The School Health Program by the United Nations Educational, Scientific and Cultural Organization (UNESCO) was also launched in 2010 to promote health activities in schools [21]. In 2012, the National Integrated Development Association Pakistan launched the Health and Nutrition Program to provide awareness about mother and child care and to educate about childhood diseases and other epidemics through active community participation [22]. In 2015, the Prime Minister of Pakistan initiated the PM’s National Health Program to provide people of lower socioeconomic status with free medical treatment [23]. In 2016, the Al Khidmat Foundation and the Maternal and Child Health Center initiated 1800 free mHealth camps all over Pakistan to train mothers on maintaining their own and their children’s health [24]. In 2017, Save the Children, an NGO, also participated to save children from disease by training lady health workers (LHWs) from different regions of Pakistan to educate young mothers and to minimize health issues [25]. The School Health Program for Pakistan commenced in 2017 to promote physical activities for children in schools, to control the consumption of snacks and junk food, and to increase the consumption of fruits and vegetables for a sound body and mind [26].

### Approach and Contributions

All the awareness efforts and education programs mentioned above were time limited to a few days or weeks. Similarly, online child health awareness and health promotion sessions are held globally, but they are of limited duration and participants usually have to pay for attending these sessions. The proposed EasyDetectDisease app, conversely, provides a long-term solution based on the following features:

- Symptoms detection
- Diseases awareness
- Nutrition awareness
- Promotion of breastfeeding
- Explanatory tutorials of live patients
- Video guidelines about diseases by pediatric health experts.

EasyDetectDisease provides a means to mothers for diagnosing the early symptoms of infectious diseases in their children. This kind of early detection of symptoms can prevent serious attacks of diseases. The app provides complete awareness about the most frequently occurring diseases in children under the age of 5 years. The EasyDetectDisease app would be a free-of-cost service, made available through governmental health agencies and app stores.

## Methods

### Overview

This section describes the main design stages of the EasyDetectDisease app development. We started off by collecting health care–related data from different websites and local health institutes. The app was designed and developed based of the collected data. Next, its usage was evaluated by a sample group of end users. Here, we explain these stages one by one.

### Data Collection

We gathered data on the diseases highlighted in the WHO and UNICEF websites and by The Children’s Hospital (CH) and the Institute of Child Health Multan (ICHM) in Pakistan. After gathering the data, 3 pediatric health experts from CH and ICHM examined the data for coherency and accuracy. The collected data were then sorted according to the instructions of experts and inserted into the app after final approval of the doctors.

### App Design

**Figure 5:**
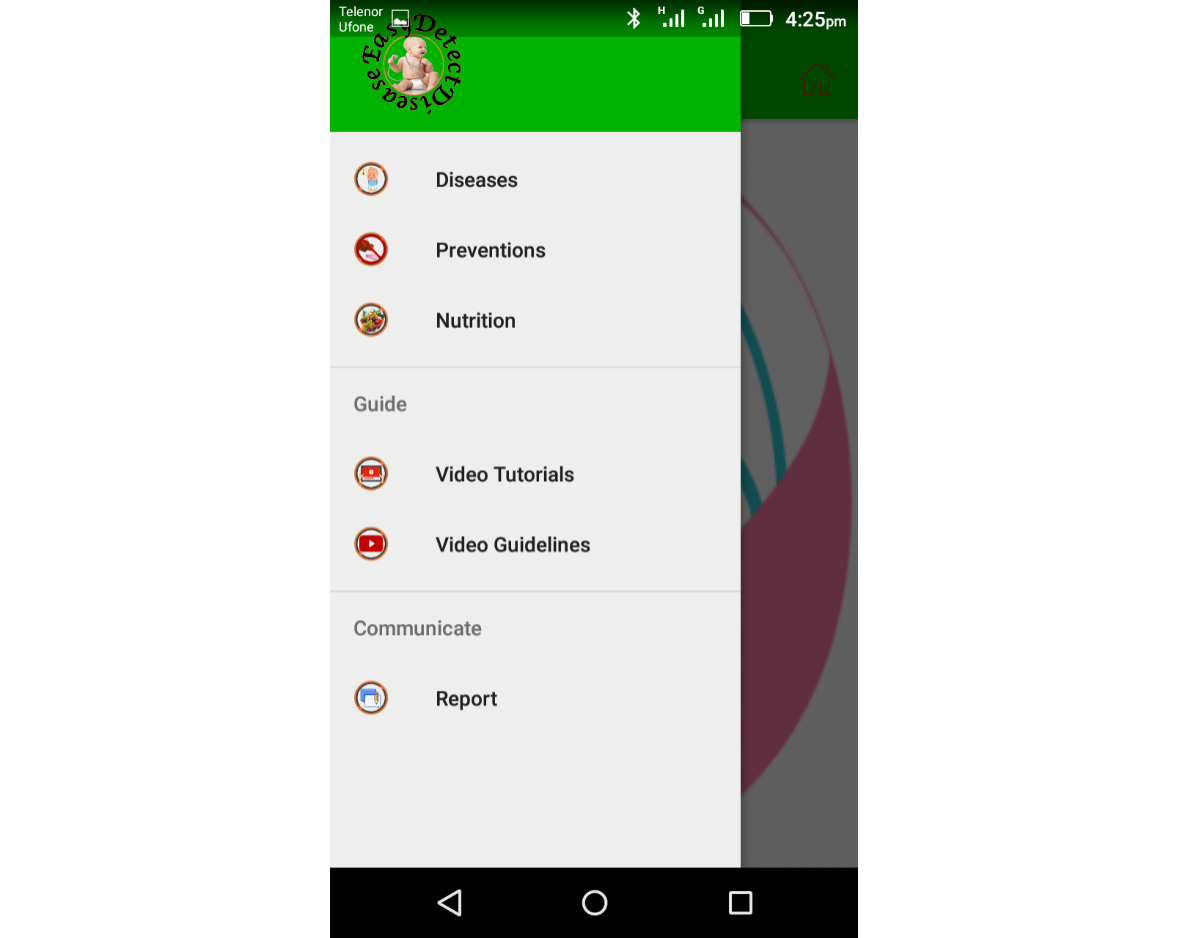
Modules of the EasyDetectDisease app in English.

**Figure 6:**
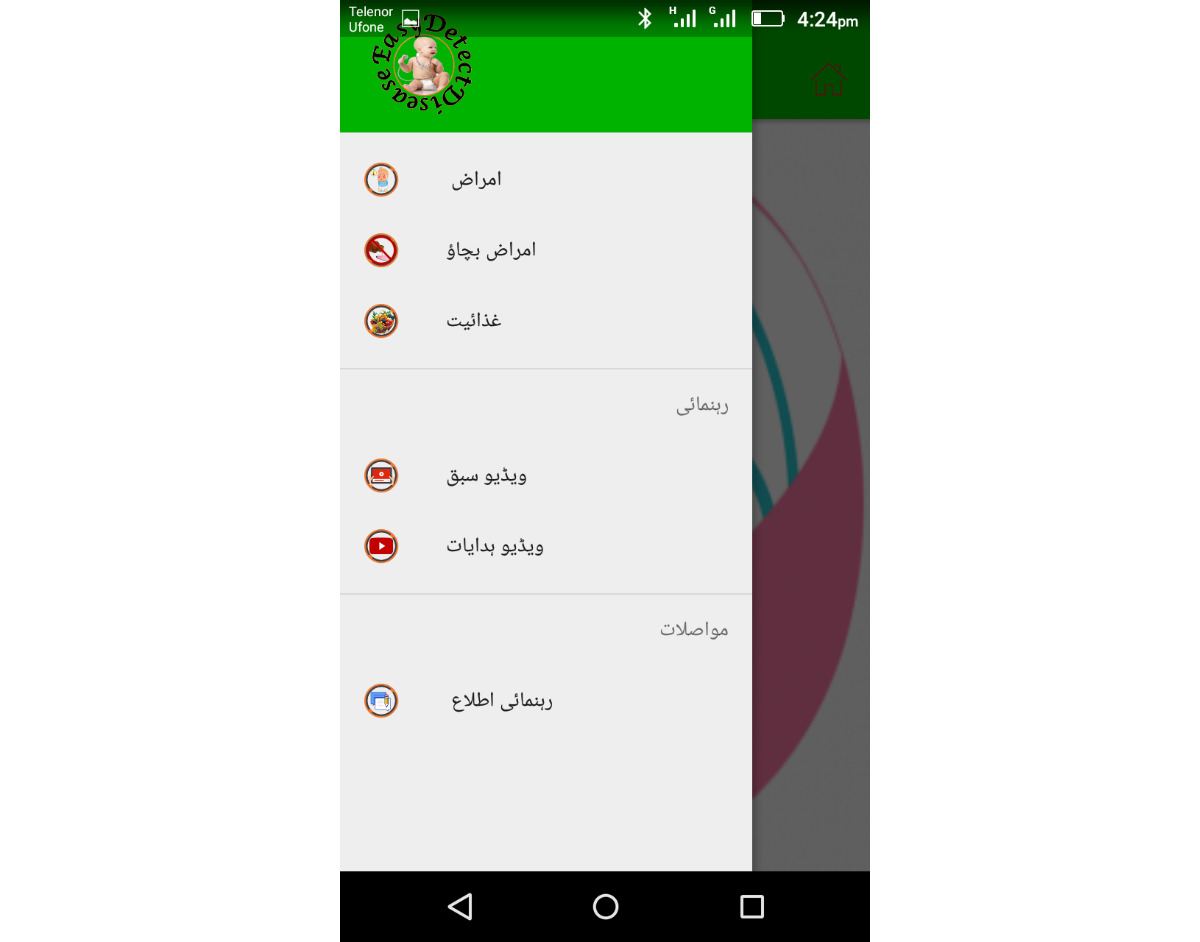
Modules of the EasyDetectDisease app in Urdu.

The EasyDetectDisease app consists of 7 modules: diagnostic test, diseases, prevention, nutrition, video guidelines, video tutorials, and report, as depicted in Figures 5 and 6. Each 1 of these 7 modules has its own functionality and purpose. The EasyDetectDisease app is bilingual and provides content in the Urdu language for Pakistani users and English for international users. The user-friendly interface has been developed to facilitate both literate and illiterate mothers in Pakistan.

According to the UNESCO data, Pakistan ranked 135th (out of 150) in literacy in 2016 [27]. According to the latest economic survey of Pakistan, the literacy rate of Pakistan has declined by 2%, from 60% in 2015 to 58% in 2017 [28]. Female literacy rate in Pakistan is 49%, which is very low compared to male literacy rate [28]; therefore, the app also provides text to speech (TTS) for mothers who cannot read Urdu but can understand it.

#### Diagnostic Test

The diagnostic test module allows the mother to detect the disease from its early symptoms. The module detects the symptoms of the sick child by asking some questions from the mother while showing her pictures of sick children.

**Figure 7:**
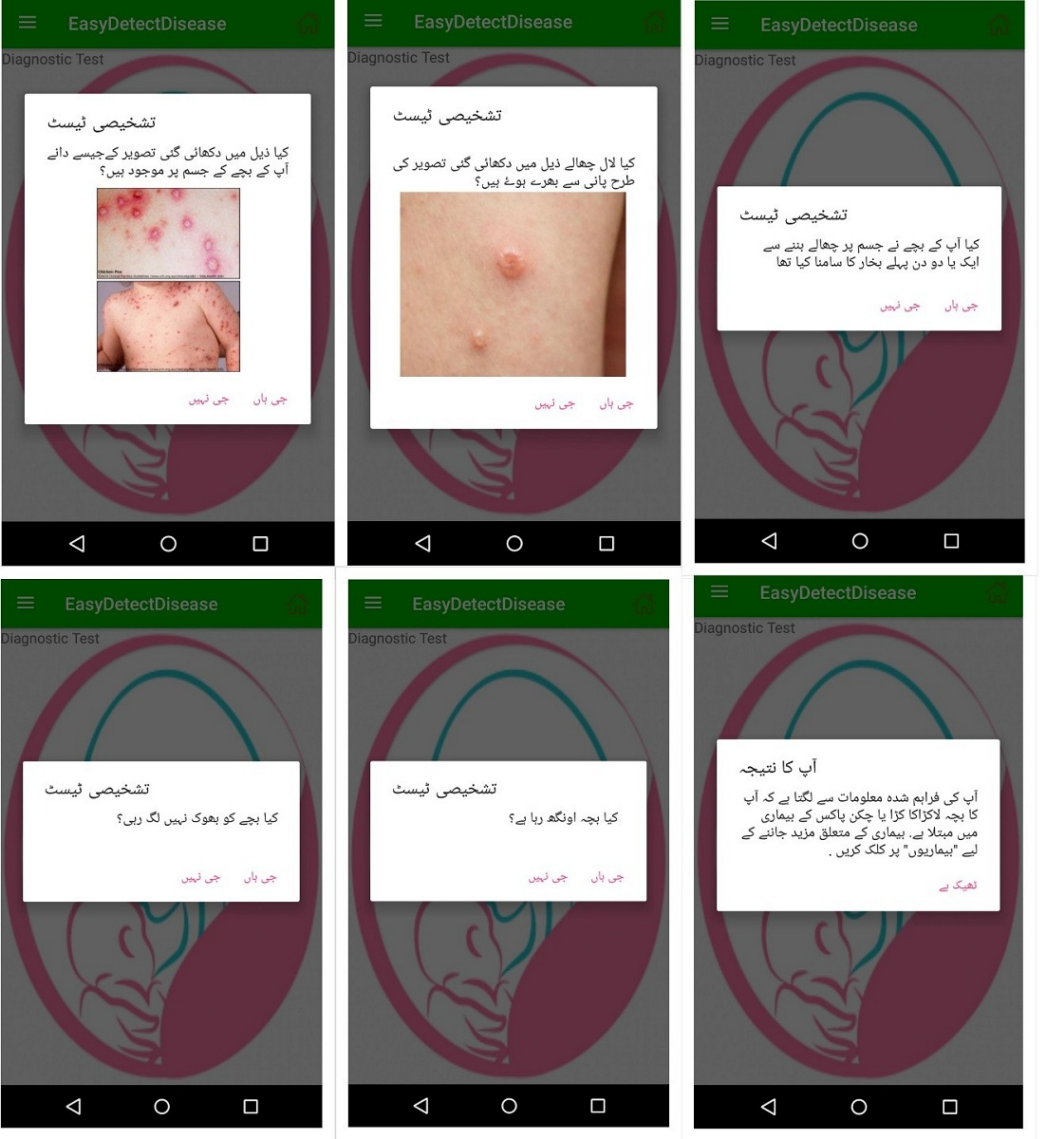
Diagnostic test interface of the EasyDetectDisease app in Urdu.

**Figure 8:**
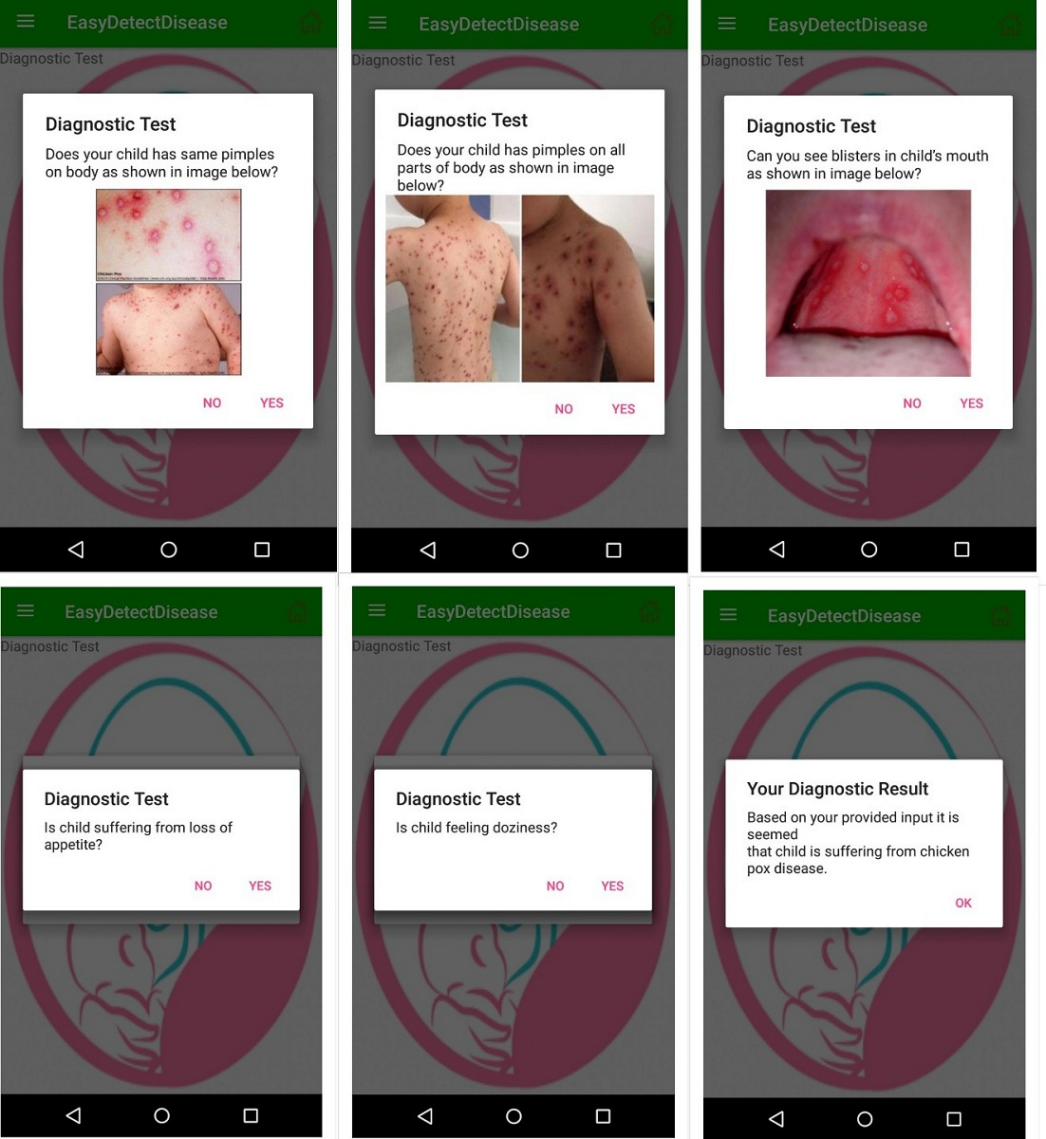
Diagnostic test interface of the EasyDetectDisease app in English.

The text of these questions is also read by the TTS. This module provides easy navigation between interfaces, and the foremost task for the mother is to select the language, and then, the user interface guides her to further navigate the app. The diagnostic test interface in Urdu and English is shown in Figures 7 and 8, respectively.

#### Diseases

**Figure 9:**
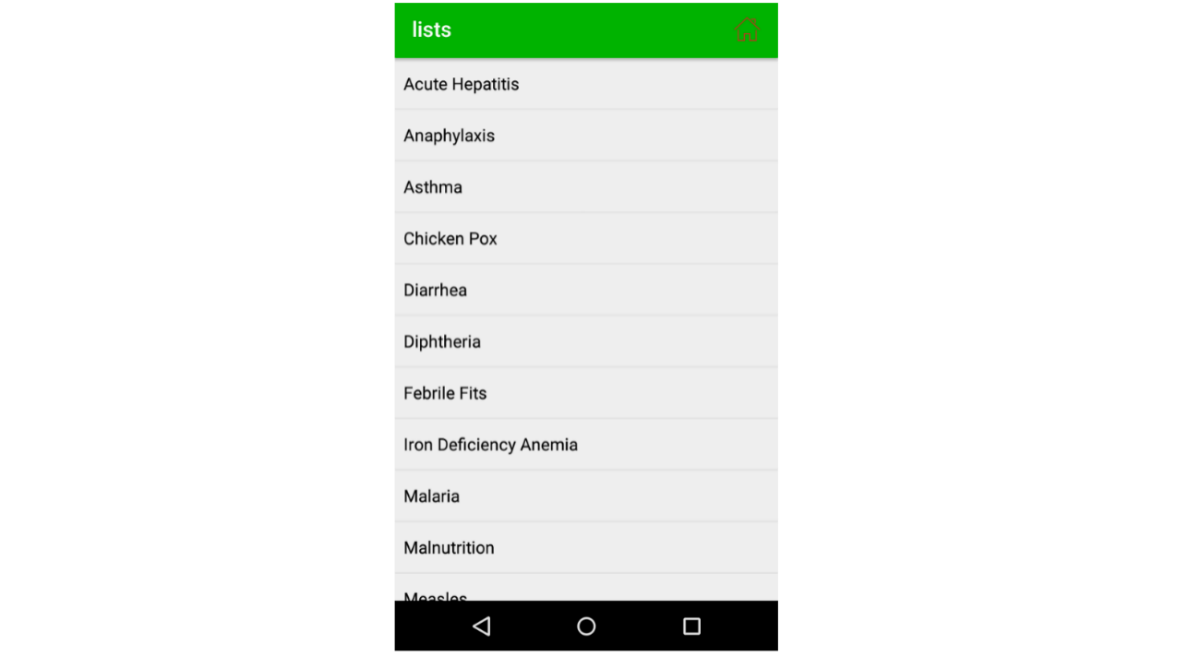
List of diseases.

**Figure 10:**
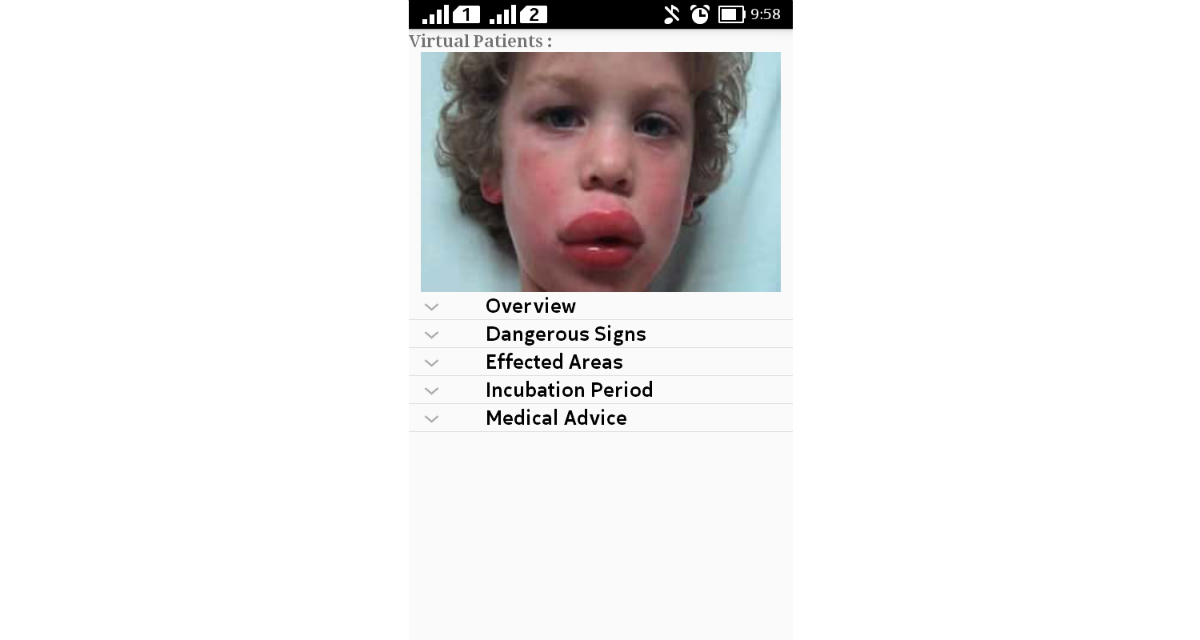
Disease description.

This module provides a detailed description of infectious diseases and some noninfectious diseases, their spread, incubation period, causes, child care at home, and medical advices. For example, if a child is suffering from anaphylaxis and his/her mother chooses anaphylaxis from the list of diseases as shown in Figure 9, the EasyDetectDisease app will show her a virtual patient to highlight the symptoms, give an overview of the disease, indicate the signs of danger and affected areas, incubation period of disease, and medical advice (see Figure 10).

#### Prevention

**Figure 11:**
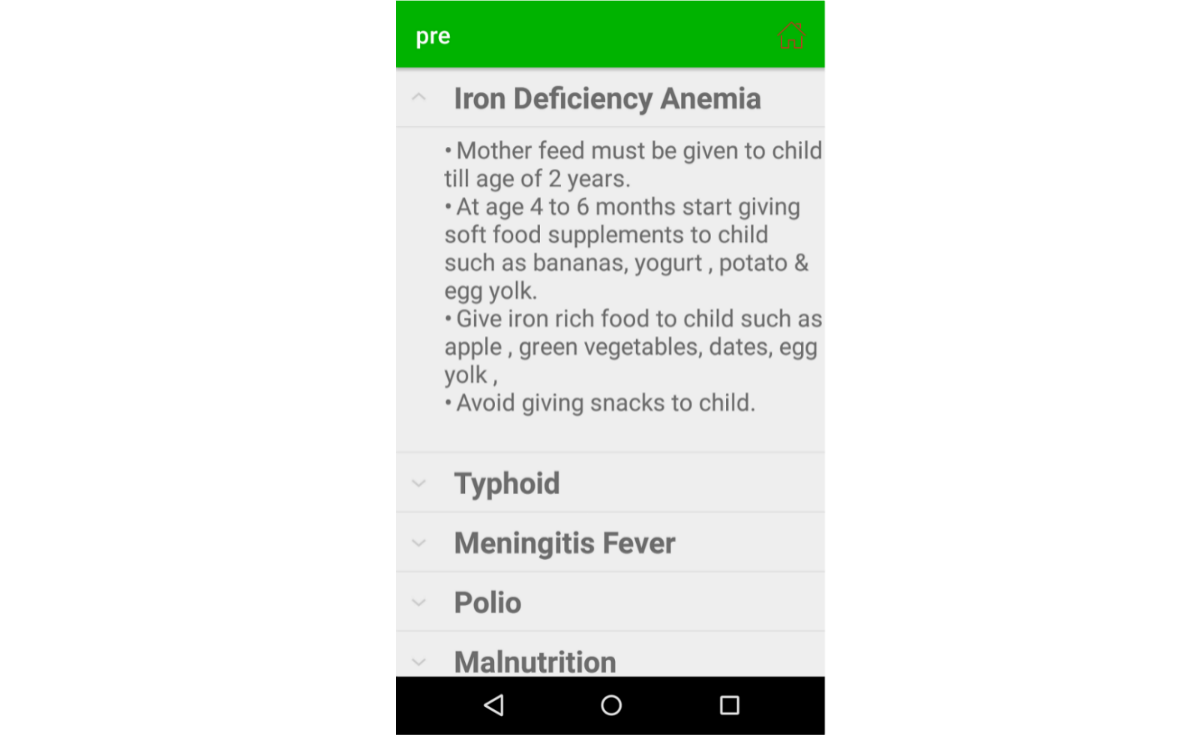
Disease prevention in English.

**Figure 12:**
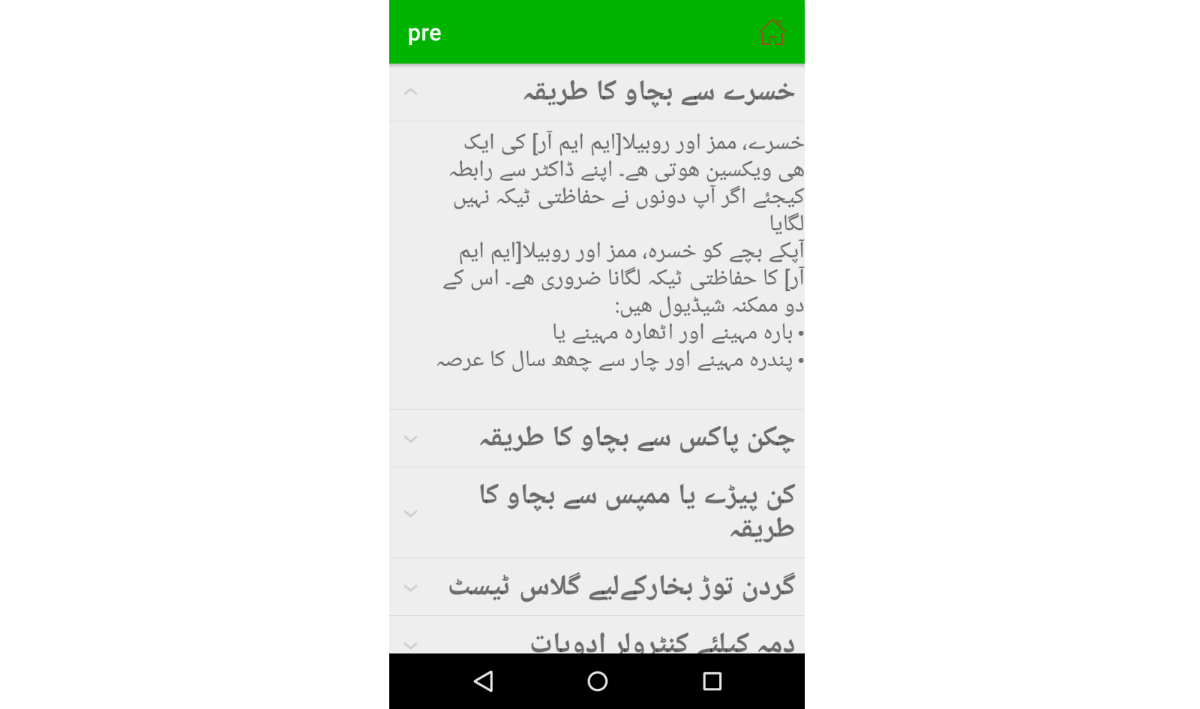
Disease prevention in Urdu.

The prevention module provides the mother with guidelines on how to prevent common diseases such as acute hepatitis, anaphylaxis, asthma, chicken pox, diarrhea, diphtheria, febrile fits, iron deficiency anemia (IDA), malaria, measles, meningitis, mumps, pertussis, tetanus, and urinary tract infection; the do’s and don’ts when the child is suffering from a certain disease; guidelines on how to maintain cleanliness; and information on the kinds of medicine that should be avoided during specific infectious diseases and can result in emergency situations (eg, death). For example, Figure 11 shows the prevention instructions for patients with IDA according to age; it highlights iron-rich foods and instructs to avoid junk food to prevent the severity of IDA. If the child is suffering from measles, the EasyDetectDisease app educates the mother on the use of the measles using measles, mumps, rubella (MMR) vaccine for prevention, and it defines the possible schedule of MMR vaccine so that the child can be cured from measles (see Figure 12).

#### Nutrition

**Figure 13:**
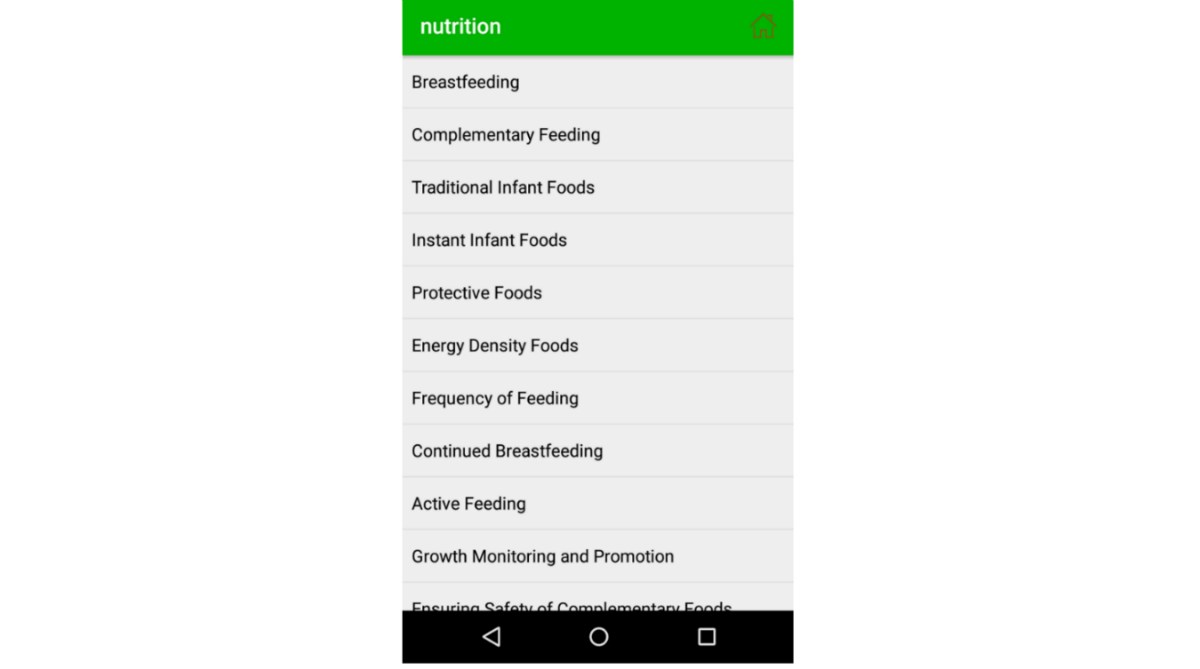
Nutritional guide in English.

**Figure 14:**
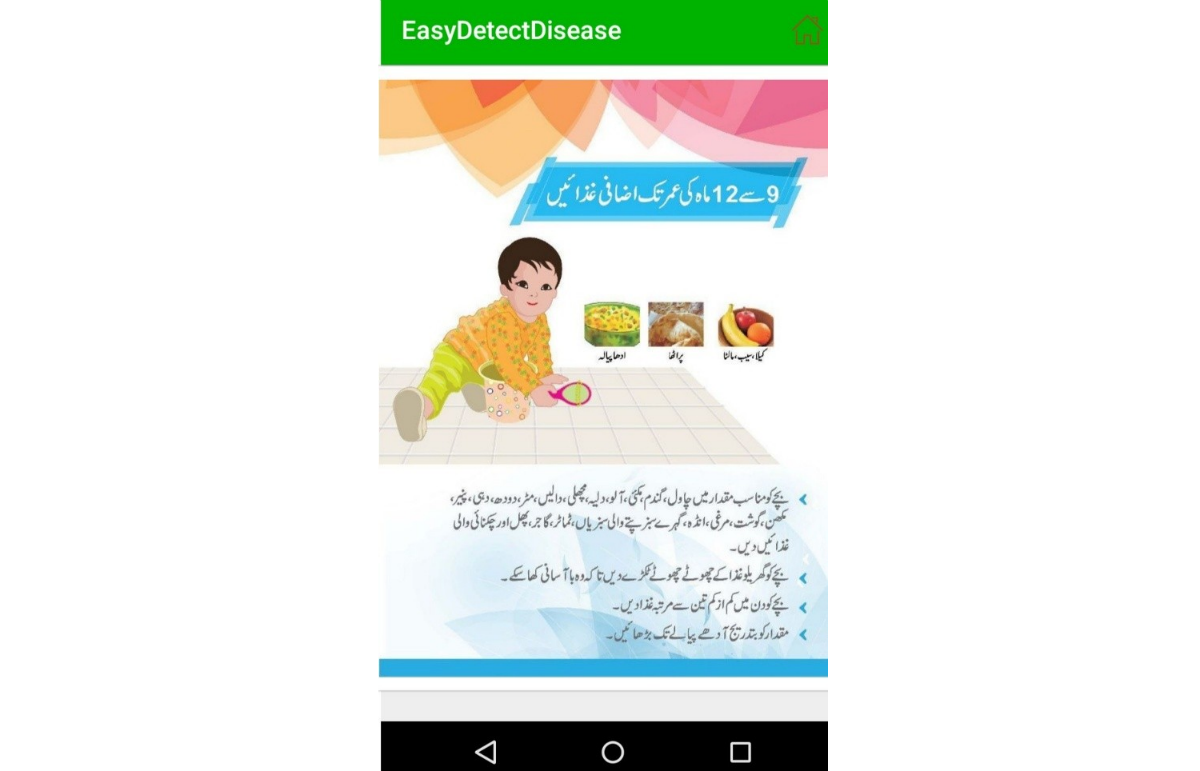
Nutritional guide in Urdu.

This module provides the nutritional guidelines to mothers. It contains dietary instructions for pregnant mothers and a nutritional guide for breastfeeding mothers (ie, feeding guidelines for newborn babies and feeding instructions for babies aged 6 to 9 months, 9 to 12 months, or 12 to 24 months). It provides details on complementary feeding, traditional infant foods, instant infant foods, protective foods, and energy-dense foods, and it instructs to monitor the growth of the child by using measuring scales. The topics of nutritional guidelines are listed in Figure 13, and Figure 14 shows the nutritional instructions for a child aged 9 to 12 months.

#### Video Guidelines

**Figure 15:**
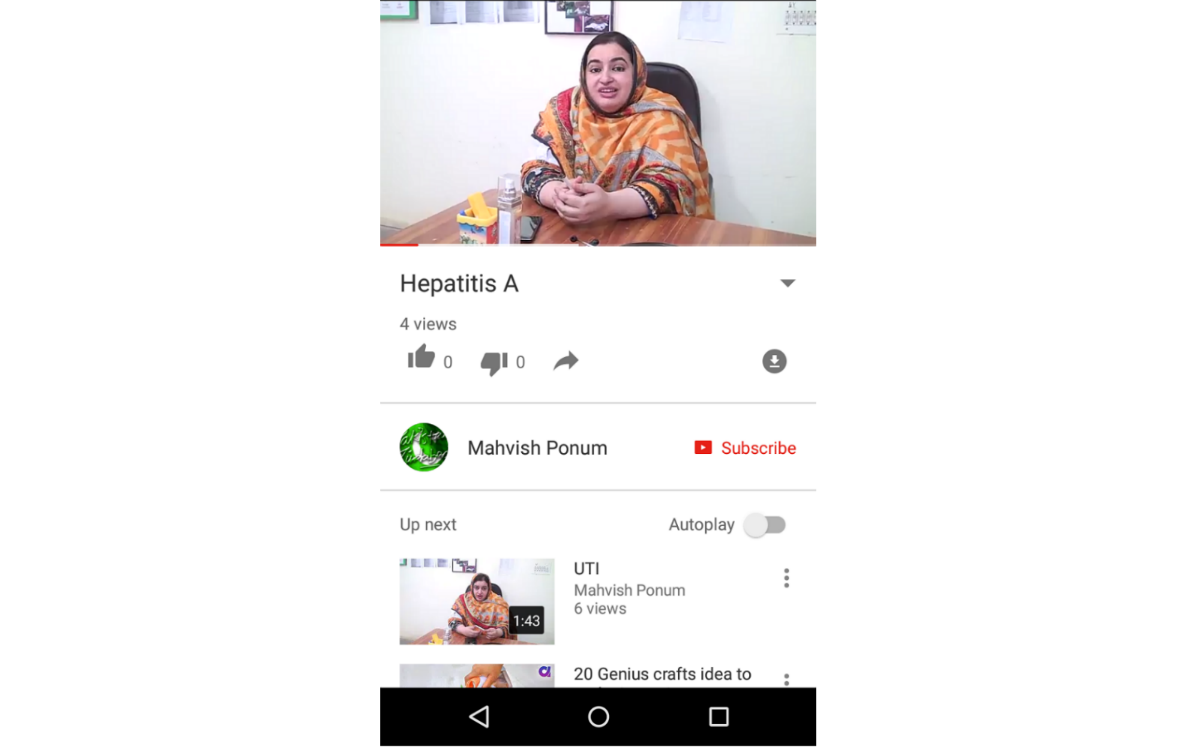
Video guidelines.

The video guidelines module consists of instructional videos recorded by pediatric health experts from CH and ICHM. In these videos, doctors educate mothers about the symptoms and prevention of diseases and provide clinical advice. For example, if a child is suffering from acute hepatitis, the pediatrician highlights the signs and symptoms of acute hepatitis before disease attack and after disease attack, causes and risk factors of acute hepatitis, its incubation period, nutrition during the disease period, prevention methods, care at home, and medical advice (see Figure 15).

#### Video Tutorials

**Figure 16:**
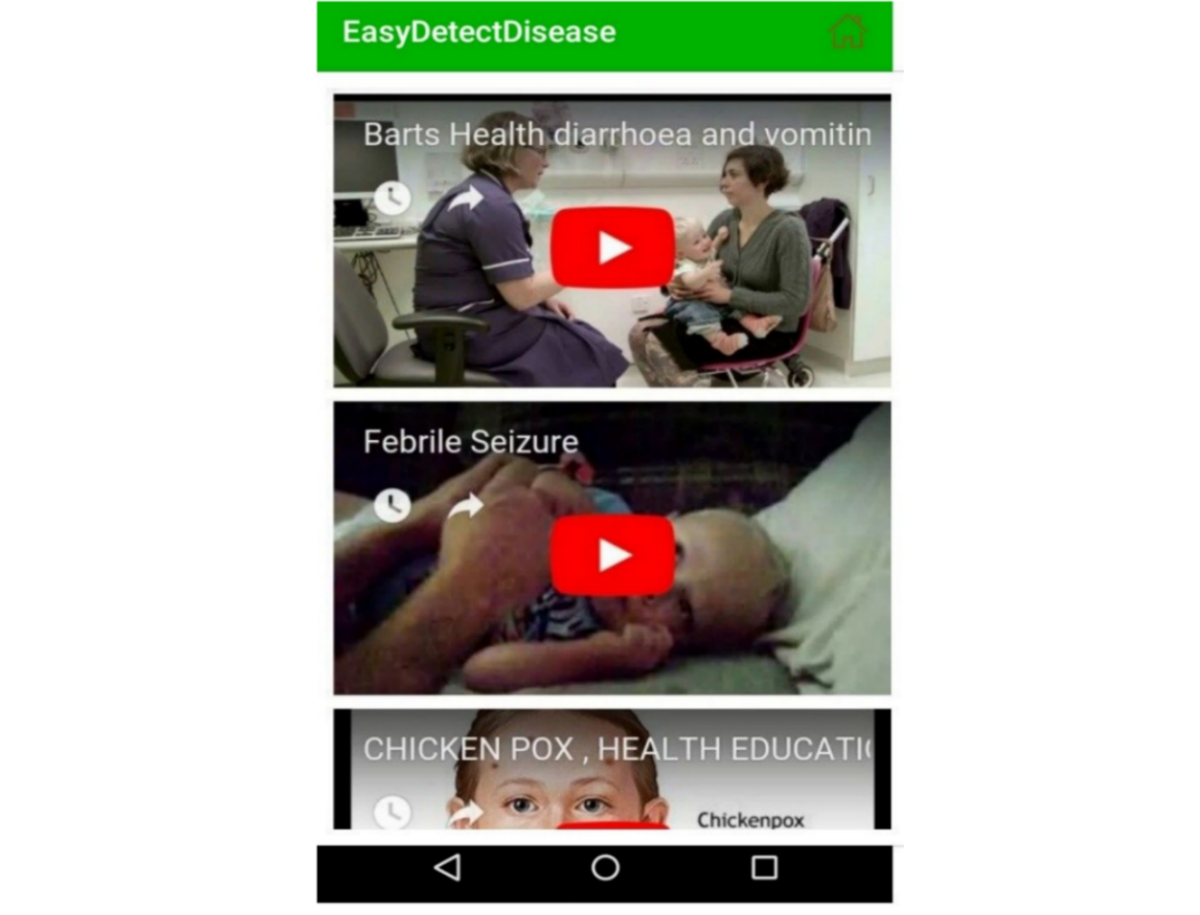
Video tutorials.

This module contains the recorded videos of actual patients affected by various diseases to practically teach mothers about the symptoms of diseases. This module also contains videos on how to make oral rehydration solution and samoji, how to use a nebulizer, etc. Figure 16 shows a list of videos about child patients with diarrhea, febrile seizure, chicken pox, and many more diseases. When a mother clicks on a specific disease, the complete guide related to that disease is provided to her by showing the affected children.

#### Report

This module allows mothers to report all those diseases that are not described well in the app or are difficult to understand. This module is primarily based on an algorithm that monitors the reports from users about diseases. If the algorithm gets 3 reports about a specific disease, then it automatically replaces the content of that disease with easier to understand material to facilitate enhanced comprehension.

### Usability Evaluation

**Figure 17:**
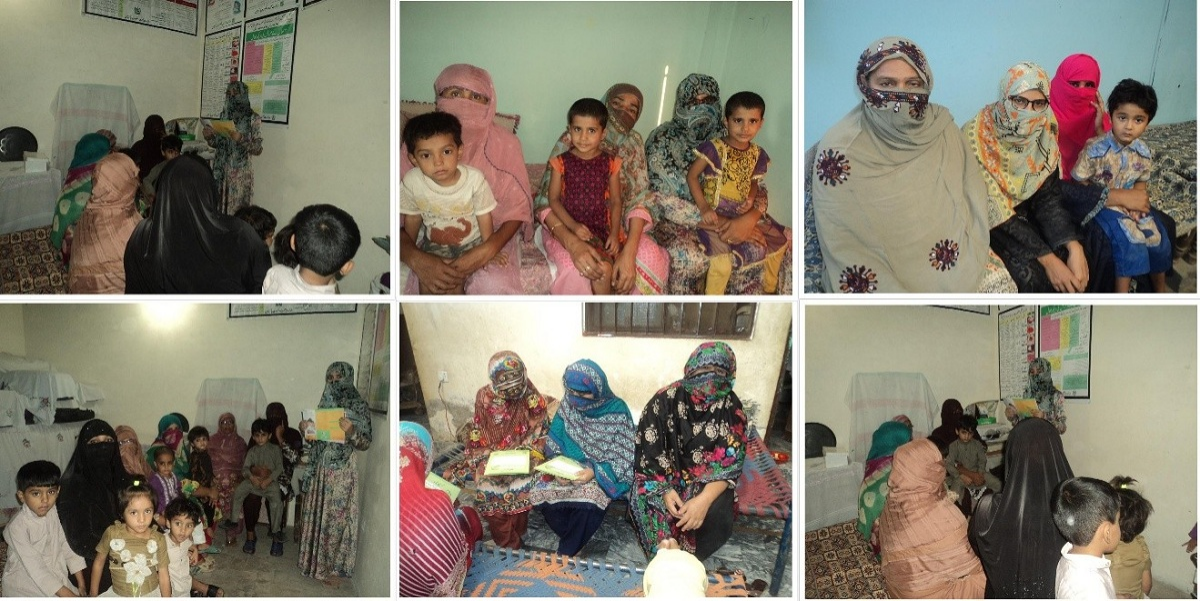
Mobile health sessions.

Overall, 2 health sessions were conducted in 2 different communities with the help of LHWs to evaluate the app’s usability (see Figure 17).

A total of 30 mothers were recruited from 2 different communities since the recommended number of participants for a usability evaluation is at least 10 [29]. According to the record kept by the LHWs, 30 mothers with ill children were called to attend the health sessions. None of these mothers had used the EasyDetectDisease app previously and most of them had their own smartphones. The app was installed on their cell phones and they were asked to use it. It was observed in the study that all those mothers who had a secondary level of education or higher used the app easily, without any help. Moreover, mothers with a primary level of education also performed well compared to illiterate mothers. The illiterate mothers were provided with a demonstration before they used the app. An explanatory usability session was conducted to explain the app’s features and its usage. It was observed that all the illiterate mothers learned how to use the app after the first explanatory session. Later, they were provided with the app to diagnose their children’s diseases in the second session where they used the app without any assistance.

After all the mothers became familiar with the diseases and their symptoms, which they could find in their children, they were provided a questionnaire (see Multimedia Appendix 1). The facilitator helped all mothers fill out the questionnaire. During the evaluation of the app, the facilitator recorded and observed the interaction of each mother with the app interface. She also noted their suggestions and comments regarding the app. At the end of the session, usability statements were evaluated on a scale of 3, ranging from strongly agree to disagree. In addition, 8 qualitative questions were asked to understand the practical effectiveness and benefits of the EasyDetectDisease app:

1. Does the user interface provide easy navigation?
2. Does the diseases module provide detailed knowledge of infectious diseases?
3. Does the prevention module provide necessary preventive measures to avoid diseases?
4. Does the nutrition module provide useful nutritional guidelines to mothers?
5. Does the video guidelines module provide complete details about diseases?
6. Do video tutorials contain useful information?
7. What is the app’s best feature?
8. Which feature of the app needs improvement?

## Results

This section provides details of the evaluation outcomes of the EasyDetectDisease app and the participants’ feedback.

### Evaluation Outcomes

The mothers of 30 ill children used the app and diagnosed the disease their child had. The ratio of the most and least common diseases was derived from the diagnostic test. The percentage of disease occurrence is shown in Table 3.

The highest number of cases were reported for diarrhea; this was diagnosed in 30% (9/30) of the children. The second major disease was chicken pox (6/30, 20%). Similarly, pertussis (4/30, 13%) and typhoid (4/30, 13%) were diagnosed as the third major diseases. The least common diseases were febrile fits and mumps (1/30, 3% for each).

After diagnosing the diseases, mothers were advised to explore the details of the disease and to follow the guidelines. They read about the symptoms and preventive measures of the disease, nutritional guidelines, and medical advice, and followed the video guidelines of the pediatric health expert. In the next meeting, each mother was asked, “What kind of health improvement did you notice in your child after using the EasyDetectDisease App?”. Most of the mothers answered that they followed all of the instructions of the app, and that they noticed that their child recovered quickly without any visible signs of weakness in the body. They did not find any dietary deficiency in their children after following the nutritional instructions.

### Participants’ Feedback

This subsection provides the results of the diagnostic module usability questionnaire and qualitative feedback from the mothers.

#### Diagnostic Module Usability Questionnaire Results

The diagnostic module questionnaire results were very positive. All participants diagnosed diseases accurately based on the symptoms. Most responses were recorded as 1 (strongly agreed). All participants strongly agreed to the statement “I liked using the EasyDetectDisease App.” All mothers found the app to be easily navigable, easy to use, and easy to understand. Very few mothers faced navigation errors because of their unfamiliarity with the app and touchscreen phones, but in the second health session, they were all able to navigate through the app.

#### Qualitative Feedback

When asked about the positive and qualitative aspects of the EasyDetectDisease app, mothers generally liked its simplicity and ease of use. One mother commented that using the app required no training as it was really easy to use. Most of the mothers reported that the app provides easy health guidelines. One mother said:

App does not need any skill to start, as I just started the app and it itself guided me what to do.

Many mothers were of the view that the disease diagnosis does not require much time as the app quickly diagnoses the disease after getting the symptoms. One mother said:

I liked the reporting feature as it automatically converts the text into easy to understand visuals.

One mother suggested that this app should be available as an built-in feature in all smartphones.

**Table 3:** Percentage of disease occurrence.

Diseases	Frequency, n (%)
Chicken pox	6 (20)
Diarrhea	9 (30)
Febrile fits	1 (3.3)
Iron deficiency anemia	2 (6.7)
Measles	3 (10)
Mumps	1 (3.3)
Pertussis	4 (13.3)
Typhoid	4 (13.3)
Total	30 (100)

## Discussion

### Principal Findings

Development of a mobile app that can be used in a cross-cultural environment with low resources is challenging, as it requires iterative testing and adaptation. The EasyDetectDisease app has been developed and updated iteratively based on feedback from end users.

The EasyDetectDisease app provides a user-friendly approach for disease diagnosis, description, symptoms, prevention, and nutrition, medical advices, tutorials featuring ill patients, and video guidelines by the pediatric health experts to promote in-home child health care. Our goal of teaching illiterate mothers about infectious diseases common in children under the age of 5 years was achieved successfully since their progress can be judged from the 2 pilot health sessions during which they used the app without any assistance. All mothers appreciated easy navigation, effectiveness, free-of-cost availability, and usefulness of EasyDetectDisease. Moreover, all mothers found the app to be very useful for their child care, and all of them agreed to use it if made publicly available.

To support the varying levels of education among mothers, many ideas were discussed (eg, adding more diseases, easiest interfaces, hint screens for illiterate mothers, and more video guidelines) that would be helpful for future implementations. Mothers with a low level of education preferred to have the app in the local languages of Pakistan. Awareness among mothers can play a vital role in reducing the mortality rate of children under 5 years. If EasyDetectDisease is made available at governmental public health agencies across Pakistan, there would be a need to educate and train mothers on some of its functionalities via governmental mHealth sessions or mHealth programs for its adaptation.

### Limitations

The EasyDetectDisease app’s current version is in its initial state, and it only contains limited and the most common infectious diseases that each child may face in his/her childhood. This app does not contain directions on dosage or medicine that may lead to death. This app is not an alternative for any medical procedure. In fact, this is an educational app that can be used to train mothers to diagnose the illness of their child at home and to immediately provide first aid by reading or listening to the guidance instructions. The awareness among mothers can prove to be the best contribution to reducing the mortality rate of children under 5 years.

Information on more diseases and additional education topics related to breastfeeding promotion, pregnancy guidelines for safe and healthy births, and noninfectious diseases will be added in the next version of the app. This app is bilingual, but in future implementations, more local languages will be added to enhance the acceptance of EasyDetectDisease in all parts of Pakistan.

## Appendix

Multimedia Appendix 1 Mortality ratios and usability questionnaire.

## Abbreviations

CH: Children’s Hospital
IDA: iron deficiency anemia
LHW: lady health workers
mHealth: mobile health
MMR: measles, mumps, rubella
NGO: nongovernmental organization
TTS: text to speech
UNESCO: United Nations Educational, Scientific and Cultural Organization
UNICEF: United Nations International Children's Emergency Fund
WHO: World Health Organization
